# Prospective, randomized, double-blind, placebo-controlled phase IIa clinical trial on the effects of an estrogen-progestin combination as add-on to inpatient psychotherapy in adult female patients suffering from anorexia nervosa

**DOI:** 10.1186/s12888-018-1683-1

**Published:** 2018-04-10

**Authors:** Georgios Paslakis, Stefanie Maas, Bernd Gebhardt, Andreas Mayr, Manfred Rauh, Yesim Erim

**Affiliations:** 1 Department of Psychosomatic Medicine and Psychotherapy, University Hospital Erlangen, Schwabachanlage 6, 91054 Erlangen, Germany; 2 Center for Clinical Studies, Krankenhausstraße 12, 91054 Erlangen, Germany; 3 Department of Medical Informatics, Biometry and Epidemiology, Friedrich-Alexander University, Universitätsstrasse 22, 91054 Erlangen, Germany; 4 Department of Pediatrics and Adolescent Medicine, University Hospital Erlangen, Loschgestraße 15, 91054 Erlangen, Germany

**Keywords:** Anorexia nervosa, Eating disorder, Estrogen, Sex hormone, Hormone replacement treatment, Neuropsychology, Cortisol, Ghrelin, Leptin, Randomized controlled trial, Psychotherapy, Appetite regulation, Neuropsychological performance, Sexual hormone

## Abstract

**Background:**

There is a need for novel treatment approaches in anorexia nervosa (AN). While there is broad knowledge with regard to altered appetite regulation and neuropsychological deficits in AN patients on the one hand, and the effects of estrogen replacement upon neuropsychological performance in healthy subjects on the other, up to now, no study has implemented estrogen replacement in AN patients, in order to examine its effects upon AN-associated and general psychopathology, neuropsychological performance and concentrations of peptide components of the hypothalamus-pituitary-adrenal (HPA) axis and within appetite-regulating circuits.

**Methods:**

This is a randomized placebo-controlled clinical trial on the effects of a 10-week oral estrogen replacement (combination of ethinyl estradiol 0.03 mg and dienogest 2 mg) in adult female AN patients. The primary target is the assessment of the impact of sex hormone replacement upon neuropsychological performance by means of a neuropsychological test battery consisting of a test for verbal intelligence, the Trail making test A and B, a Go/No-go paradigm with food cues and the Wisconsin Card Sorting Test. Secondary targets include a) the examination of safety and tolerability (as mirrored by the number of adverse events), b) assessments of the impact upon eating disorder-specific psychopathology by means of the Eating Disorder Examination Questionnaire (EDE-Q) and the Eating Disorder Inventory-2 (EDI-2), c) the influence upon anxiety using the State-Trait-Anxiety Inventory (STAI), d) assessments of plasma cortisol levels during a dexamethasone-suppression test and appetite-regulating plasma peptides (ghrelin, leptin, insulin, glucose) during an oral glucose tolerance test and, e) a possible impact upon the prescription of antidepressants.

**Discussion:**

This is the first study of its kind. There are no evidence-based psychopharmacological options for the treatment of AN. Thus, the results of this clinical trial may have a relevant impact on future treatment regimens. Novel approaches are necessary to improve rates of AN symptom remission and increase the rapidity of treatment response. Identifying the underlying biological (e.g. neuroendocrinological) factors that maintain AN or may predict patient treatment response represent critical future research directions. Continued efforts to incorporate novel pharmacological aspects into treatments will increase access to evidence-based care and help reduce the burden of AN.

**Trial registration:**

European Clinical Trials Database, EudraCT number 2015–004184-36, registered November 2015; ClinicalTrials.gov Identifier: NCT03172533, retrospectively registered May 2017.

## Background

Anorexia nervosa (AN) is characterized by restrictive eating behaviors, an intense fear of gaining weight, body image disturbances and amenorrhea. AN is a serious and complex mental illness [1, 2] and is often characterized by a chronic and disabling course [3, 4]. In fact, one-third of adult AN patients show poor outcome due to an enduring illness with relevant consequences on all aspects of quality of life [5, 6]. Recent follow-up studies failed to provide evidence that treatments can influence long-term results [7, 8]. The public health-related costs are significant [9]. Established psychotherapeutic treatments (e.g. cognitive behavioral therapy, interpersonal psychotherapy) are being implemented in the treatment of AN [10, 11]. There are just few empirically-supported treatments for AN. Watson et al. [12] performed a detailed review of randomized controlled trials (RCTs) in AN published between 1980 and 2011; the authors found that no treatment has been shown to be superior than the others, with results primarily focused on short-term findings. According to a recent systematic review of third-wave behavior therapies for the treatment of AN, none of the third-wave therapies meet criteria for an empirically-supported treatment for AN; thus, patients with AN should be treated with cognitive behavioral therapy, with interpersonal therapy considered an alternative [13]. The NICE guidelines published in 2017 include specialist supportive clinical management (SSCM), individual eating disorder-focused cognitive behavioral therapy (CBT-ED) and Maudsley Anorexia Nervosa Treatment for Adults (MANTRA) as the three recommended treatments for adult AN [14]. At the same time, there are no evidence-based psychopharmacological options for the treatment of AN, thus hindering the implementation of medication in AN patients [15].

### Neuroendocrinology of anorexia nervosa

Both low food intake and/or high energy expenditure due to extensive training disrupt the hypothalamic signals to the pituitary gland. These disruptions lead to multiple endocrine and metabolic changes in AN that are generally considered adaptive mechanisms for the conservation of energy. The suppression of the menstrual cycle in females is one of the consequences.

#### Hypothalamus-pituitary-gonadal (HPG) axis and its implications in anorexia nervosa

Gonadotropin-releasing hormone (GnRH) from the hypothalamus binds to receptors in the pituitary gland leading to the production of luteinising hormone (LH) and follicle-stimulating hormone (FSH). In turn, LH and FSH in females act primarily on the ovaries to produce estrogen and to regulate the menstrual and ovarian cycle.

Female patients with AN show a dysregulated hypothalamus-pituitary-gonadal (HPG) axis; a hypothalamic amenorrhea is a common finding in females suffering from AN [16]. Hypothalamic amenorrhea results from the suppression of the pulsatile secretion of GnRH. The pulsatile secretion of LH in AN is also disrupted, showing either LH pulses of low amplitude (similar to pre- or early pubertal girls), or increases only during sleep [17], resulting in estrogen deficiency [18, 19]. This state of hypogonadotropic hypogonadism has detrimental effects on several organ systems including a reduced bone mineral density and infertility [20, 21].

#### Hypothalamus-pituitary-adrenal (HPA) axis in anorexia nervosa

In humans, cortisol is the major biologically active natural glucocorticoid, the production of which is tightly regulated by the adrenocorticotropic hormone (ACTH), secreted from the anterior pituitary gland in response to the corticotropin-releasing hormone (CRH) produced by the hypothalamus.

CRH concentrations in the cerebrospinal fluid of AN patients have been shown to be higher than normal, both during the active phase of the disease and after short-term weight recovery [22]. Sustained hypercortisolemia in AN has been repeatedly detected in a great number of studies [23–28]. Hypercortisolemia may be severe, with urinary free cortisol excretion reaching levels observed in patients with Cushing’s disease or severe depression [29]. Hypercortisolemia in AN is considered to be an adaptive mechanism that maintains euglycemia during states of reduced energy availability [26].

High cortisol levels in AN patients predict a low percentage of extremity lean mass [30] and low bone mineral density [25, 26]. High cortisol levels in AN patients also predict the severity of psychopathological symptoms independently from the body mass index (BMI) [31]. Research has found elevated cortisol levels in AN patients to be normalized after weight recovery [32, 33]. However, exposure to high levels of cortisol stimulates weight gain with central fat accumulation when there is sufficient substrate. When AN patients gain weight, preferential truncal fat gain is seen. Trunk fat accumulation appears to be most severe in those patients with the greatest increases in urinary free cortisol concentrations [34].

#### Appetite-regulating hormones in anorexia nervosa

Fasting plasma levels of the orexigenic peptide ghrelin have been reported to be raised in underweight AN patients [35] and tend to normalize with recovery of body weight, parallel to the progressive increase in weight during restoration treatments [36]. The suppressant effect of oral glucose administration on plasma ghrelin was significantly blunted in females with AN [37], while the food-induced suppression of circulating ghrelin was almost absent in the study by Nedvidkova et al. [38]. Increased ghrelin secretion stimulates the secretion of CRH, which in turn stimulates the secretion of ACTH and cortisol [27, 39]. CRH seems to play an additional important role in feeding regulation acting as a satiety stimulator [32] and has been associated with weight loss [40]. Ghrelin and cortisol concentrations in AN have been found to be positively associated [41].

The concentrations of leptin, the key regulator in the brain to decrease food intake and increase energy expenditure, are lower than normal in the plasma and the CSF of patients with AN [35]. Reduced secretion of leptin has been considered responsible for a lifetime history of amenorrhea and subnormal serum levels of LH [42]. Physiologically, leptin and insulin have stimulatory effects and ghrelin has inhibitory effects on GnRH secretion [43], thus amplifying the vicious circle of HPG axis dysregulation in AN patients. Low leptin levels are also considered potentially responsible for low hypothalamus-pituitary-thyroid axis function, increased HPA axis function and osteopenia in AN ([42]. Alterations of leptin resolve with the recovery of body weight [44]. In long-term recovered AN patients, both plasma and CSF levels of the hormone appear to be similar to those in healthy controls [45]. Longitudinal studies have shown that leptin concentrations and secretion rate progressively increase as weight is regained and in those cases with a too rapid weight restoration, they reach values disproportionally higher than normal [46].

Additionally dysregulated central and peripheral modulators of appetite in AN patients have been extensively reviewed by Tortorella et al. [47].

### Neuropsychological performance in AN

Many authors have reported neuropsychological impairments (e.g. deficits in learning and memory tasks) in AN patients. Candidate intermediate phenotypes for AN are cognitive inflexibility (poor set shifting), weak central coherence and social-emotional processing difficulties (e.g. [48–53]). Neuropsychological impairments may be considered the result of lack of energy availability due to the drastically reduced food intake. In addition, the above mentioned alterations in the HPG as well as the HPA axis may play a role.

Compelling evidence indicates that glucocorticoids are essential for cognitive functions due to influences on brain regions crucial to attention, learning and memory, such as the prefrontal cortex, hippocampus and basolateral amygdala. The hippocampus appears to be particularly sensitive to increased levels of glucocorticoids, possibly because it is dense with glucocorticoid receptors [54, 55]. High cortisol levels in AN patients have been associated with structural brain abnormalities and weak cognitive performance [56, 57], as well as learning and memory impairments [58]. As in animal models, prolonged hypercortisolemia was associated with reduced hippocampal volume measured by magnetic resonance imaging (MRI), which was partially reversed following cortisol decrease during treatment [59]. The duration of illness (and of hypercortisolemia) seems to be a crucial factor, as the degree of hypercortisolemia could not be associated to cognitive dysfunctions in non-chronic AN patients [60, 61]. In general, such changes resolve with weight rehabilitation during inpatient treatment [62], although in some cases, circumscribed persistent deficits are found before and after short-term weight rehabilitation [33, 63].

Estrogen is mainly associated with verbal memory [64–66]. The growth of parahippocampal grey matter volumes during puberty is contingent upon increasing estrogen levels [67]. In a well-controlled study, healthy young females were found to perform better on verbal fluency tasks during the mid-luteal phase (high estrogen concentration) than during the early follicular phase (low estrogen concentration) of their menstrual cycle [65]. Abnormal menstrual function and estrogen depletion are associated with the presence of cognitive deficits, including verbal and working memory and retrieval in AN patients [66, 68]. Apart from AN, cognitive impairments have also been demonstrated in other estrogen-deficient states such as the menopause [66].

### Estrogen replacement therapies

Estrogens and progesterone (P4) are sex steroid hormones predominantly found in females. Females produce three major forms of estrogen including estrone (E1), 17ß-estradiol (E2) and estriol (E3), with E2 being the most bioactive and potent estrogen under normal physiological conditions [69]. During the menstrual cycle there are two peaks of E2 secretion, one just before ovulation and a smaller one during the mid-luteal phase. Progesterone levels remain low during the follicular phase; there is one peak in secretion during the mid-luteal phase [69].

Oral contraceptives (OCs) are the most widely employed form of reversible contraception. To date, almost 200 million women, at ages ranging from puberty to premenopause have taken various formulations of OCs [70].

Combined OCs contain both a synthetic estrogen and progestin. Ethinyl estradiol (EE) is a synthetic form of the endogenously circulating E2 and is by far the most commonly used form of estrogen in OCs. EE is more potent than the endogenous E2 [71]. In contrast, the progestin component varies with each OC brand. There are currently several generations of progestins that differ in their biological properties. For example, the second generation progestins are often derived from testosterone and are thus more androgenic compared to the third generation progestins, whereas the newest progestins are anti-androgenic [72, 73].

#### The effect of estrogen replacement on neuropsychological performance in healthy females

Sex steroid hormones are primarily produced by the ovaries in females, but may also be synthesised by the brain [69]. The central nervous system is a major target of sex steroid hormone action and shows a high expression of sex hormone receptors [74]. There are a number of overlapping regions of expression of sex hormone receptors including the forebrain and hippocampus. This fact supports a role of sex hormones on functions other than the reproduction system [75, 76]. Indeed, sex hormones have been implicated in brain development, mood and cognition. Numerous studies suggest that estrogens, especially E2, have neurotrophic effects, improve cerebral blood flow, and promote synaptic plasticity and myelination [77]. Several major neurotransmitter systems are modulated by estrogens, including the dopaminergic and glutamatergic pathways [78, 79]. In addition, acute applications of E2 have antioxidant [80] and anti-apoptotic properties [81].

Based on the current literature, estrogen is generally understood to have a positive effect on cognitive performance (particularly in tasks like verbal memory) [82, 83]. One of the most reliable findings in this area is an improvement in verbal memory with OC use [84–86].

With regard to visuospatial ability, the majority of studies found no significant difference between OC users and non-users [86]. As far as other domains are concerned, studies are inconclusive with regard to attention, working memory and executive function (judgement, planning and control) or show no convincing evidence of altered social and emotional cognition and psychomotor performance with OC use [86]. Overall, the interpretation of these results is impeded by limitations such as lack of control for confounders and lack of blinding. In the study by Gogos [84], users of OCs (high synthetic estrogen/progestin levels) performed similarly to mid-luteal phase (high E2/P4) non-users of OCs and better than non-users in the early follicular phase (low E2 and P4), suggesting that high levels of sex hormones have a positive effect on higher-order cognition, especially memory. In addition, several studies have reported effects of OCs on brain structure and activation using fMRI [87–89].

In an extensive review, Toffoletto et al. [90] provided a systematic summary on the current state-of-the-art with regard to fMRI studies on the effects of ovarian and exogenous sex steroid hormones on emotional and cognitive functioning in different cohorts, among others in healthy naturally cycling females, female users of OCs and females exposed to hormonal interventions. The authors reported remarkable differences regarding brain reactivity between menstrual cycle phases, as well as between users of OCs and naturally cycling females [90].

However, there are also studies showing little or no effect of OCs on higher-order cognition [85, 91, 92]. Apparently, influential effects are task- and context-dependent.

#### Estrogen replacement therapy in AN

There is only a limited number of studies available examining the effects of estrogen replacement in AN patients, while most studies focus on changes in bone mineral density following treatment (e.g. [93–95]. Brain regional hypometabolism in the posterior cingulate cortex of females with AN was attenuated towards normal with low-dose testosterone replacement [96]. In the study by Misra et al. [97], the authors examined the effect of estrogen replacement upon AN-associated psychopathology and found that estrogen replacement in adolescent AN patients reduces trait anxiety independent of weight changes, although it did not directly impact eating attitudes and behaviors, body shape perception or state anxiety. Possible associations to cortisol levels or appetite-regulating peptides were not investigated in this study by Misra et al. [97].

While there is broad knowledge with regard to altered appetite regulation and neuropsychological deficits in AN patients on the one hand and the effects of estrogen replacement upon neuropsychological performance in healthy subjects on the other, up to now, no study has implemented estrogen replacement in adult female AN patients to examine its effects upon neuropsychological performance, AN-associated and general psychopathology and concentrations of peptide components of the hypothalamus-pituitary-adrenal (HPA) axis and within appetite-regulating circuits.

It is hypothesized that:

- Significant improvements in neuropsychological performance will be observed in the estrogen-progestin-treated compared to the placebo group.
- Significant improvements in AN-associated psychopathology will be observed in the estrogen-progestin-treated compared to the placebo group.
- Significant improvements towards restitution/normalization of disrupted appetite-regulating circuits will be observed in the estrogen-progestin-treated compared to the placebo group (see “methods”).

## Methods

### Design

This prospective, randomized, double-blind, placebo-controlled, monocentre phase IIa clinical trial will compare outcomes for estrogen-progestin-treated and placebo-treated female AN patients upon a series of outcome parameters (see below) during inpatient AN-specific psychotherapy. Furthermore the study will investigate the safety and tolerability of the estrogen-progestin treatment compared to placebo. Patients in the verum group will receive a combination of ethinyl estradiol 0.03 mg and dienogest 2 mg over a period of ten weeks. The protocol is in accordance with the SPIRIT guidelines [98]. This clinical trial has been approved by the Federal Institute for Drugs and Medical Devices (Bundesinstitut für Arzneimittel und Medizinprodukte, BfArM, Bonn, Germany), the German Competent Authority for medicinal products and medical devices, and received the approval of the independent Ethics Committee of the Friedrich-Alexander-Universität Erlangen-Nürnberg, Germany. Monitoring of the study will be carried out by a qualified member of the Center for Clinical Studies, Erlangen, Germany according to the Good Clinical Practice (GCP) guidelines.

### Study population

The study population will be adult female patients suffering from AN with a clinical indication for rigorously standardized inpatient behavioural therapy treatment.

The following inclusion criteria are defined: 1. diagnosis of AN according to the Diagnostic and Statistical Manual of Mental Disorders (DSM V, APA 2013) or subsyndromal AN (lack of a diagnostic symptom according to DSM V), 2. BMI ≥ 13 kg/m^2^ and ≤ 18.5 kg/m^2^, 3. age ≥ 18 yrs. and ≤ 45 yrs., 4. patients able to provide written informed consent.

A series of exclusion criteria have been determined: 1. a known hypersensitivity to the active compounds or to other components of the study drug, 2. one or more contraindications for the use of hormonal contraception: smoking ≥20 cigarettes/day; acute venous thromboembolic disease or increased risk; known hereditary or acquired predisposition for venous thrombosis, e.g. activated protein C (APC) resistance (including factor V Leiden mutation), antithrombin III deficiency, protein C deficiency, protein S deficiency; risk for arterial thromboembolism (diabetes mellitus with vascular sequelae, severe hypertonus, severe dyslipoproteinemia); known hereditary or acquired predisposition for arterial thrombosis, e.g. hyperhomocysteinemia and anti-phospholipid antibodies (anticardiolipin antibodies, Lupus anticoagulants); cerebrovascular disease (past cerebral infarction or prodromal states such as transitory ischemia attacks); past migraine with focal neurological symptoms; liver disease or pancreatitis; Dubin-Johnson syndrome and Rotor syndrome; known porphyria; known or suspected sexual hormone sensitive tumours; unresolved vaginal bleeding, 3. a present severe depressive episode (major depression) according to the DSM V; a mild or moderate depression does not constitute an exclusion criterion (and will be controlled for in the statistical analyses). The intake of antidepressants at a stable dose for a minimum of 14 days prior to study inclusion does not constitute an exclusion criterion, 4. past or present alcohol or drug abuse, 5. severe psychiatric disorders (axis I) according to the DSM V (such as bipolar affective disorder or schizophrenia) in addition to AN, 5. suicidality, 6. known diabetes mellitus, 7. severe somatic comorbidity or organ dysfunction that is not compatible with intake of the study drug, 8. use of hormonal depot compounds (injectable drugs, implants), or hormonal intrauterine pessaries during the last four weeks before the screening visit, 9. pregnancy, 10. breastfeeding during the last six months before the screening visit, 11. current intake of St. John’s wort (CYP3A4 inductor), rifampicin, cholestyramine, ampicillin or tetracycline; at least five half-life cycles of the respective compound must have passed since last intake.

### Recruitment

All participants are inpatients at the University Hospital Erlangen, Germany, Department of Psychosomatics and Psychotherapy. During an outpatient presentation before hospitalization, patients are screened by a medical professional with regard to inclusion and exclusion criteria and are asked to participate. Study inclusion takes place upon admission to our inpatient ward.

Female patients who gave consent to take part in the study and are already taking an OC will be asked to discontinue their contraceptive pill for a minimum of 28 days, in order to ensure a wash-out phase. Subjects will be asked to take additional contraceptive measures (at least two) to prevent pregnancy. Pregnancy tests in the course of the trial are scheduled; a pregnancy will immediately lead to discontinuation of study participation. All subjects will be examined by a staff member of the Department of Gynecology at the University Hospital Erlangen, as to ensure the absence of contraindications against the intake of an OC. A continuous intake of the study drug over a period of ten weeks is in accordance with common clinical OC practice, when due to medical reasons or patient’s preference menstruation should be omitted. Compliance to treatment will additionally be measured by interim measures of plasma sex hormones.

### Sample size

A total of *n* = 50 subjects (25 patients per treatment arm) will be included in the analyses.

### Randomization and blinding

The study will be performed in a randomized double-blind design. Neither participants, nor medical staff, investigators or outcomes assessors have insight into patients' allocation to each of the two study arms. Blinded study medication will be provided by the Hospital Pharmacy of the University Hospital. Subjects will be randomly assigned to the treatment arms of either verum or placebo at a 1:1 ratio. Prior to study initiation, a randomization list was generated by an external independent person using the nQuery software (Statistical Solutions Ltd., Cork, Ireland) and a block randomization design (block size *n* = 6). The assignment of medication to study participants is carried out by two persons, one acting as the operator and the second acting as the checker. Study drug intake will be supervised by the study staff.

### Data collection

A series of data during seven visits (V1 to V7) are collected during this trial.

#### Eating disorder-specific and general psychopathology

All subjects will be asked to fill in the Eating Disorder Examination-Questionnaire (EDE-Q) and the Eating Disorder Inventory-2 (EDI-2). The EDE-Q [99] evaluates eating disorder psychopathology in the past 28 days. It contains 22 items that have to be answered on a 7-point scale ranging from “never” to “every day”. It comprises four subscales assessing a) restraint, b) eating concern, c) weight concern and d) shape concern. Internal consistency of the German version was α = 0.97 in the validation study [100]. As this study includes inpatients whose meal intake is monitored by clinical psychologists and/or medical professionals, in order to ensure constant weight gain, the subscale “restraint” is not applicable in this study. The EDI-2 [101] is a self-report questionnaire with 91 items that assesses eating disorder psychopathology, but also inter- and intrapersonal aspects thought to be relevant for the development and perpetuation of eating disorders [101] (e.g. perfectionism, interpersonal distrust, lack of interoceptive awareness etc.). Higher scores indicate more severe psychopathology; only the total score will be considered in the analyses.

In addition, the State-Trait-Anxiety Inventory (STAI; [102]) for anxiety assessment, the Patient Health Questionnaire for the assessment of depression, (PHQ-9; [103]) and the Eating Disorder Quality of Life (EDQOL; [104]) for assessment of impairments in quality of life due to AN will be applied.

#### Blood sampling for general clinical assessment, neuroendocrinological parameters, sex hormones and appetite-regulating circuits

All subjects will undergo a dexamethasone-suppression test (DST), as an easy-to-perform test for the assessment of HPA axis activity at baseline and at the end of treatment at week 10 (EOT). In detail, 1 mg dexamethasone will be administered orally at 11.00 PM and cortisol in plasma will be assessed at 8.00 AM on the following day. Dexamethasone suppressors and non-suppressors will be considered in the statistical analyses.

In addition, an oral glucose tolerance test (OGTT) will be performed and plasma levels of ghrelin, leptin, insulin and glucose will be determined at baseline and at week 10 (EOT) to evaluate concentration changes in the course of this functional procedure.

Hormone levels of LH, FSH, estradiol, testosterone will be determined at baseline, week 2, week 6 and week 10 (EOT).

Blood samples will be obtained at baseline, at week 2 and week 10 to evaluate routine laboratory parameters (including blood count, fasting lipids, CRP, transaminases etc. for safety reasons). Interim assessments of further safety data (vital parameters, physical examination, urine pregnancy test etc.) are scheduled.

### Neuropsychological performance

In addition, the neuropsychological performance of the subjects will be assessed using a neuropsychological battery consisting of the following tests: a) a test for evaluation of verbal intelligence and speech comprehension (“Wortschatztest”, WST; [105]), b) the Trail Making Test (TMT) [106] for the assessment of executive function, attention, information processing speed and planning ability, c) the WCST [107] as a test of frontal brain function. The test examines categorization ability among different categorization options and has an excellent sensitivity with regard to disturbed problem solving and perseverations, d) a go/no-go task as a paradigm for response inhibition, thus refraining an action initiation [108]. This task was programmed with Presentation® software (Version 16.0, Neurobehavioral Systems, Inc., Albany, CA, USA) to assess impairment of inhibitory control in response to food-related and control stimuli (for a detailed description see [109]). Changes on each test will be reported.

The neuropsychological assessment will be repeated after the 10-week intervention period.

A follow-up visit six weeks after EOT is scheduled. A summary of all study procedures is given in Table 1.

**Table 1:** A synopsis of study procedures from visit 1 (V1) to visit 7 (V7)

	V1	V2	V3	V4	V5	V6		V7
	Screening	Baseline	Treatment start	Week 2	Week 6	Week 10 (EOT)		End visit (EOS) week 16
	month − 2 ± 2	day − 3 (±2)	day 1	day 15 (±3)	day 43 (±3)	day 73 (±3)		day 113 (±7)
Randomization			X					
Intake of study drug			X------>	-----------	------------	--------X		
Information about study objectives and informed written consent	X							
Inclusion and exclusion criteria	X	X						
Medical history		X						
Sociodemographic data		X						
Anthropometric data (height, weight, waist Circumference, hip circumference, BMI)		X					X	
Weight (BMI)		X	X	X	X		X	X
12-channel ECG		X					X	
Urine pregnancy test		X					X	
Vital parameters (HR, RR)		X	X	X	X		X	
Physical examination		X		X			X	
Gynecological examination		X						
Inclusion criteria and contraindications before first intake (gynecological findings, liver parameters, RR etc.)			X					
EDE-Q			X				X	X
EDI-2			X				X	X
STAI			X				X	X
PHQ-9			X				X	X
EDQOL			X				X	X
Neuropsychological test battery: verbal intelligence test, TMT A and B, Go/NoGo-test with food stimuli, WCST			X				X	
Dexamethasone-suppression test/plasma cortisol concentration (5)			X			X		
Plasma sex hormones (4)		X		X	X	X		
OGTT for the assessment of plasma ghrelin, leptin, glucose and insulin (6)		X				X		
Routine laboratory tests (1)(2)(3) and urine dipstick		X		X		X		
Assessment of concurrent medication		X	X	X	X	X	X	X
Assessment of adverse events			X	X	X	X	X	X

1 hematology: hemoglobin (Hb), hematocrit, red blood cells, white blood cells, platelets, differential blood count, coagulation parameters (Quick/ PTT)

2 clinical chemistry: sodium, potassium, urea, chloride, creatinine, bilirubin, GOT, GPT, gamma-GT, alcaline phosphatase, lipase, LDH, CRP

3 fasting lipids

4 sex hormones: LH, FSH, estradiol, testosterone

5 dexamethasone-suppression test: 1 mg dexamethasone per os at 11.00 PM followed by fasting blood collection at 08.00 AM for plasma cortisol

6 OGTT (oral glucose tolerance test): blood draw before intake of glucose suspension (0 min) as well as 30, 60, 90 und 120 min after ingestion

*TMT A and B* Trail Making Test A and B, *WCST* Wisconsin Card Sorting Test

### Statistics

The present trial is the first of its kind. Most trials have examined the effect of estrogen replacement upon bone mineral density in adolescent patients suffering from AN. Thus, in this phase IIa clinical trial, sample size cannot be calculated in the same way as in confirmatory phase III studies due to a missing valid base regarding effect sizes and variability of target parameters. However, a sample size calculation according to the results of a similar trial [97] with regard to the change in trait anxiety has been initially conducted. Following the treatment effects found in Misra et al. [97], *n* = 25 patients are needed in both groups in order to achieve a statistical power of at least 80% considering a two-sided t-test with the common significance level of 5%. With this number of patients, effect sizes (differences in means with a common SD = 1) of 0.80 can be detected (again with a two-sided t-test and significance level of 5%) with a power of 80%, e.g. at the end of treatment. Smaller effects sized of 0,60 or 0.70, could be only detected with a power of 55% or 68%. For a power of 85%, 90% and 95% effect sizes of 0.86, 0.94 or even 1.04 would be necessary.

Data of the present study may be used for future sample size calculations of confirmatory studies, as well as for the generation of new hypotheses. The intended statistical analysis follows the intention-to-treat principle. Differences between the treatment groups at baseline will be assessed using Fisher’s test for categorical variables and the non-parametric Mann-Whitney test for metric variables due to the relatively small sample size. For cross-sectional analysis of primary and secondary endpoints, adjustments for potential confounders and group differences at baseline by means of regression models will be applied. Missing outcome values will be imputed by corresponding baseline values. For longitudinal analyses, appropriate mixed models considering the longitudinal structure of data and interrelations due to random patient-specific effects will be used. Due to the robustness of this approach towards missing values, no imputation is planned for the longitudinal analyses. No interim analysis is scheduled. Effect sizes will be reported based on Cohen’s d for group differences for primary and secondary outcomes. Clinical improvements, amelioration in neuropsychological performance and restitution of hormonal aberrancies are all to be considered of clinical significance for patients with AN. The target parameters of safety and tolerability will be analysed descriptively (absolute and relative frequency with confidence intervals, mean values and medians with appropriate distributions).

### Outcome measures

The primary outcome measure is the change in neuropsychological performance based on the above described neuropsychological test battery after ten weeks of hormonal substitution with an estrogen-progestin-combination. The secondary outcome measures following the 10-week intervention are: a) incidence of treatment-emergent adverse events (AEs) (safety/tolerability) mirrored by the number of AEs (including AEs, adverse reactions [ARs], serious AEs, serious ARs and suspected unexpected serious ARs [SUSARs]), b) changes in psychopathology (EDE-Q, EDI-2, STAI, PHQ-9, EDQOL) mirrored by changes in sum scores, c) changes in cortisol mirrored by changes in plasma cortisol levels during a dexamethasone suppression test as well as changes in plasma concentrations of the appetite-regulating peptides ghrelin, leptin, insulin and glucose during an OGTT and d) changes in antidepressant medication mirrored by changes in their use.

## Discussion

AN is a disorder with high morbidity rates and a severe influence on quality of life. The disorder shows one of the highest mortality rates among all psychiatric-psychosomatic disorders. As a result of restrictive eating patterns and low BMI, neuroendocrinological circuits are regularly altered in AN patients. Indeed, several findings show a compensatory activation of the HPA axis with hypercortisolemia and a suppressed HPG axis with estrogen deficiency. Also with regard to central and peripheral concentrations of appetite-regulating peptides like ghrelin or leptin, AN patients show deviations from the norm. Moreover, it has been shown that clinical symptom amelioration and weight gain are associated with a restitution of dysregulated hormonal circuits.

There are practically no evidence-based pharmacological treatments for AN. The aim of the present trial is to investigate in a randomized, placebo-controlled manner the effects of an estrogen-progestin replacement over ten weeks on the clinical phenotype (disorder-specific and general psychopathology as well as neuropsychological performance) of AN, on the regulation of the HPA and HPG axes and appetite-regulating peptides in AN, but also with regard to safety and tolerability. The only randomized controlled studies available so far have examined the effects of estrogen replacement on bone mineral density in adolescent AN patients. Thus, the trial presented here is the first of its kind.

The results of this clinical trial may have a relevant impact on future treatment regimens in the battle against AN. Novel approaches are necessary to improve rates of AN symptom remission and increase the rapidity of treatment response. Identifying the underlying biological (e.g. neuroendocrinological) factors that maintain AN or may predict patient treatment response represent critical future research directions. Continued efforts to incorporate novel pharmacological aspects into treatments will increase access to evidence-based care and help reduce the burden of AN.
